# A leg ulcer revealing Parkes Weber syndrome in a child

**DOI:** 10.11604/pamj.2025.51.22.47960

**Published:** 2025-05-27

**Authors:** Leila Debono, Imane Laatfa

**Affiliations:** 1 Pediatric's Medical Emergency, Children's Hospital of Rabat, Rabat, Morocco,; 2 Faculty of Medicine and Pharmacy of Rabat, Mohamed V University in Rabat, Rabat, Morocco

**Keywords:** Parkes Weber, ulcer, child, arteriovenous malformations

## Image in medicine

A 13-year-old male child with no prior history presented to the pediatric emergency room for a lesion on the anterior aspect of his left leg associated with pain throughout the limb. On clinical examination, significant hypertrophy of the left lower limb was noted. In the thigh and calf of the same limb, several veins were prominent, enlarged, and followed a tortuous course. The lesion in question, located on the lower part of the medial aspect of the leg, was rounded, with loss of substance, minimally oozing, and clean, without necrosis or associated signs of ischemia. The femoral, popliteal, and pedal pulses were present and symmetrical with the contralateral pulses. The right lower limb was unharmed. The cardiac examination found no murmur or added noise, or signs of heart failure. The mucocutaneous examination did not reveal any abnormalities, notably no angiomas. A Doppler ultrasound was performed, revealing multiple arteriovenous communications. The assessment was completed by a computed tomography (CT) angiography of the left lower limb, which confirmed high-flow arteriovenous malformations with thrombosis of the left internal iliac vein and the left superficial femoral vein, consistent with Parkes-Weber syndrome. The CT scan also noted skeletal hypertrophy of the left lower limb with muscular hypertrophy and thickening of the soft tissues of the left leg with skin irregularity and loss of substance in the middle third, suggesting an ulcer. The patient was put on anticoagulant treatment with local treatment of his ulcer using 10% povidone iodine and petroleum jelly. The course was marked by an improvement in the ulcer lesion and disappearance of the pain.

**Figure 1:**
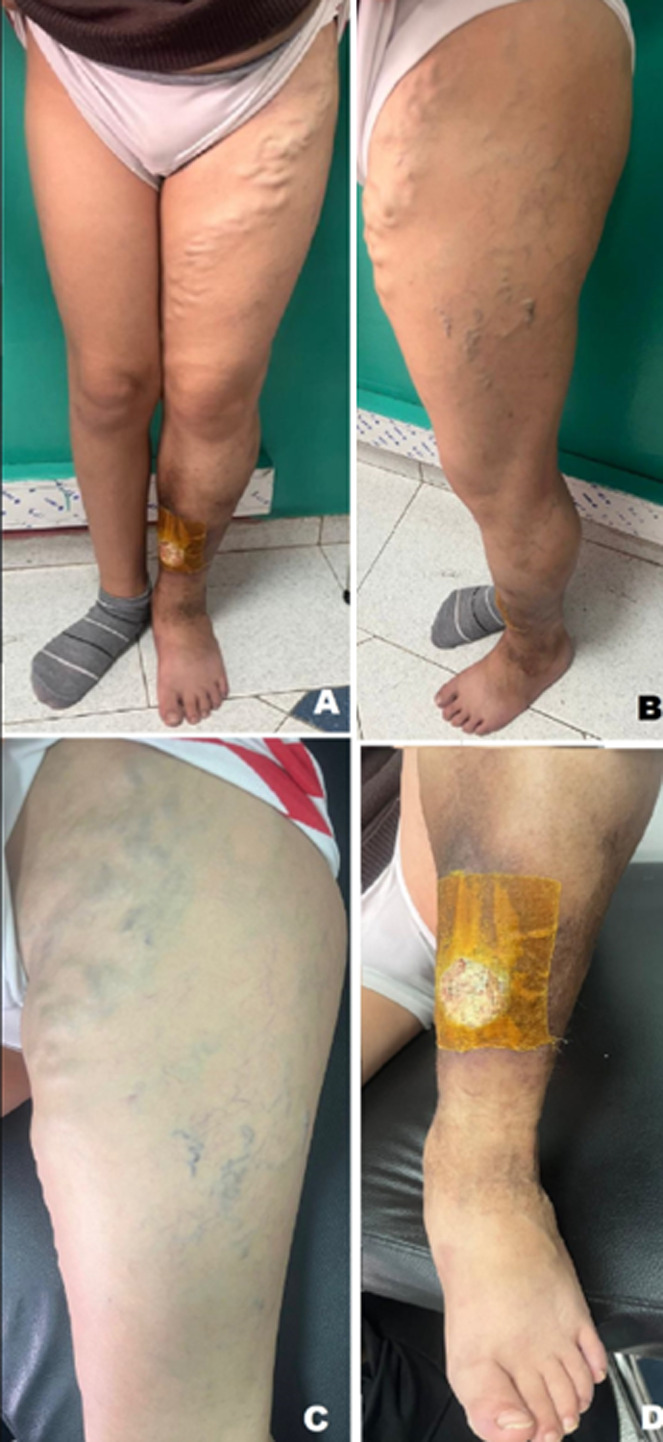
A) frontal image of both lower limbs showing significant hypertrophy of the left lower limb; B) side view of the left lower limb showing an enlarged limb with collateral venous circulation; C) frontal image of the left thigh showing prominent veins at the root and collateral venous circulation; D) frontal image of the left leg showing the ulcerative lesion in the middle third

